# Supplementary material for: Structural insight into the electron transfer pathway of a self-sufficient P450 monooxygenase
Source: Nat Commun. 2020 May 29;11:2676. doi: 10.1038/s41467-020-16500-5 (PMC7260179; doi:10.1038/s41467-020-16500-5)
Supplement: Supplementary file 1 — Supplementary information [file 41467_2020_16500_MOESM1_ESM.pdf]

Supplementary Information

Structural insight into the electron transfer pathway of a self-sufficient P450 monooxygenase

Lilan Zhang, Zhenzhen Xie, Ziwei Liu et al.

Supplementary Figures

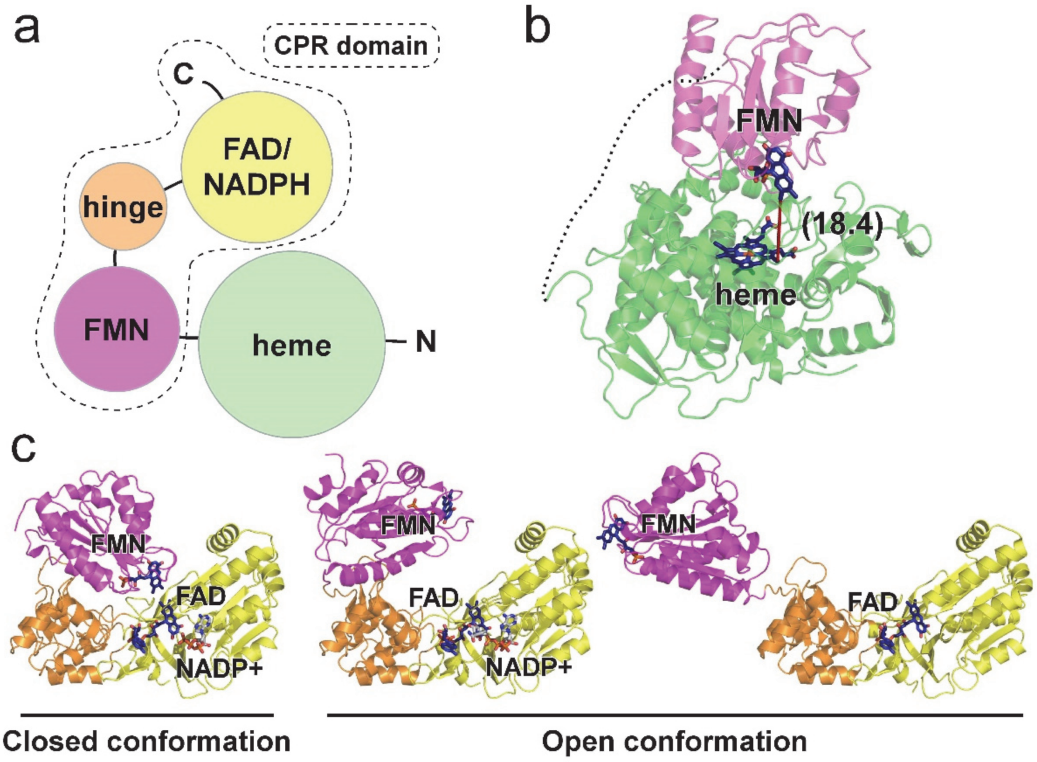

Supplementary Figure 1. Domain organization of P450BM3. (a) The overall domain arrangement of P450BM3. The CPR domain fused to the C-terminus of heme domain is indicated by dash line. (b) The complex structure of heme domain and FMN binding domain of P450BM3 (PDB ID, 1BVY)[1]. The dashed line links two domains that should be connected by a missing fragment of 20 protein residues. (c) Various conformations of mammalian CPRs. (Left) Rat CPR (PDB ID, 1AMO)[2]; (middle) ΔTGEE mutant of Rat CPR (PDB ID, 3ES9)[3]; (right) yeast-human chimeric CPR (PDB ID, 3FJO) [4]. The color scheme used to display crystal structures in (b) and (c) is the same as used in (a). Co-factors bound in each domain are labeled and shown as sticks. The edge-to-edge straight-line distance between cofactors within each complexes are shown in parentheses (unit, Å).

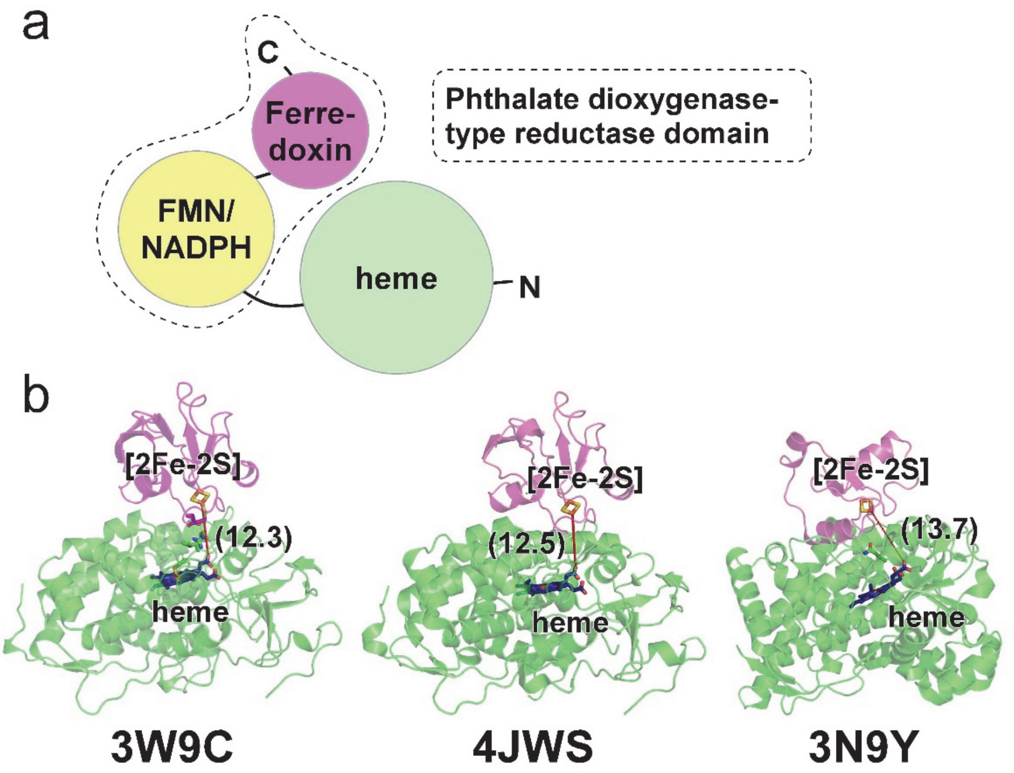

Supplementary Figure 2. Domain organization of CYP116 P450s. (a) The overall domain arrangement of CYP116 P450s. The phthalate dioxygenase-type reductase domain fused to the C-terminus of heme domain is indicated by dash line. (b) Complex structures of (left and middle) P450cam heme domain and putidaredoxin[5, 6], and (right) CYP11A1 heme domain and adrenodoxin[7]. Protein structures are presented in cartoon model with their PDB ID indicated below. The heme domains and redox transfer domains are colored in green and magenta. Co-factors bound in each domain are displayed as sticks. The edge-to-edge straight-line distance between cofactors within each complexes are shown in parentheses (unit, Å).

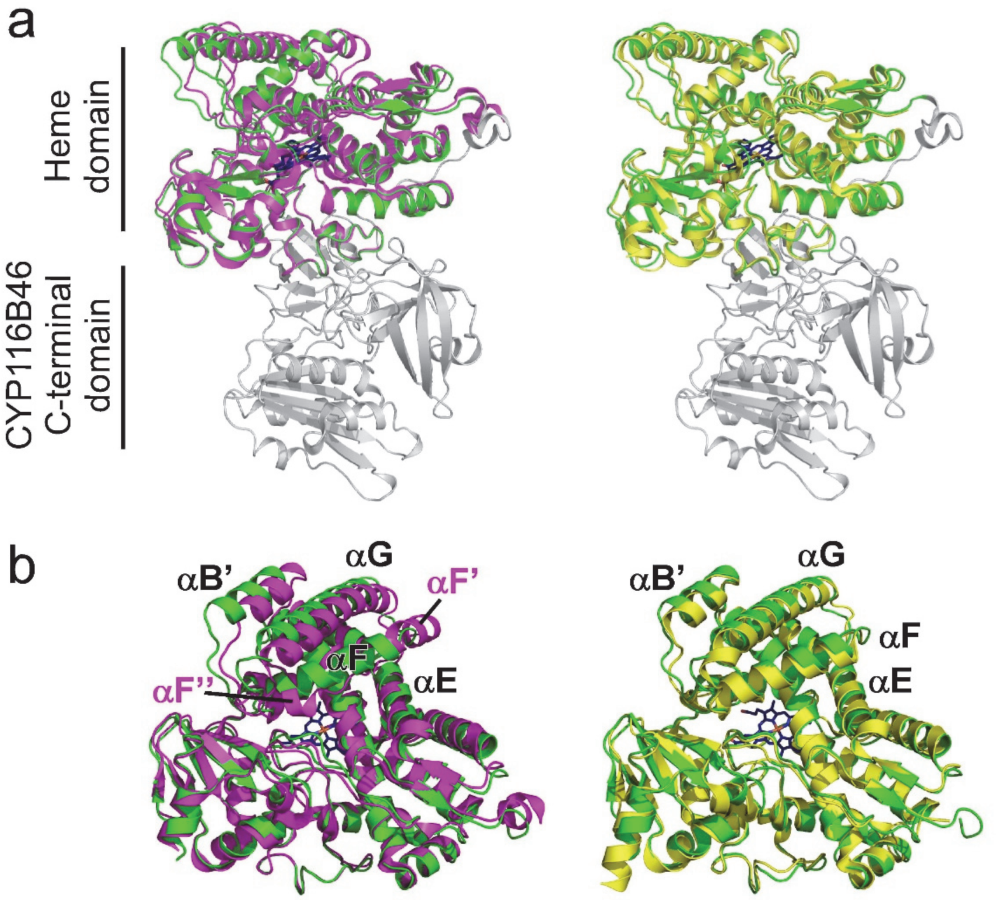

Supplementary Figure 3. Structural comparison of heme domain of CYP116B family. (a) Structural superimposition of full-length and (left) CYP116B46-N (PDB ID, 6GII)[8] or (right) CYP116B5-N (PDB ID, 6RO8)[9]. The heme domain and C-terminal domain of the full-length structure are colored in green and gray. The CYP116B46-N and CYP116B5-N are in magenta and yellow color, respectively. (b) Superimposition of heme domain from the full-length structure of CYP116B46 and (left) CYP116B46-N and (right) CYP116B5-N. Some helicases showing conformational alterations are labeled. Helix αF in full-length CYP116B46 and CYP116B5-N split into helix αF' and αF" in CYP116B46-N.

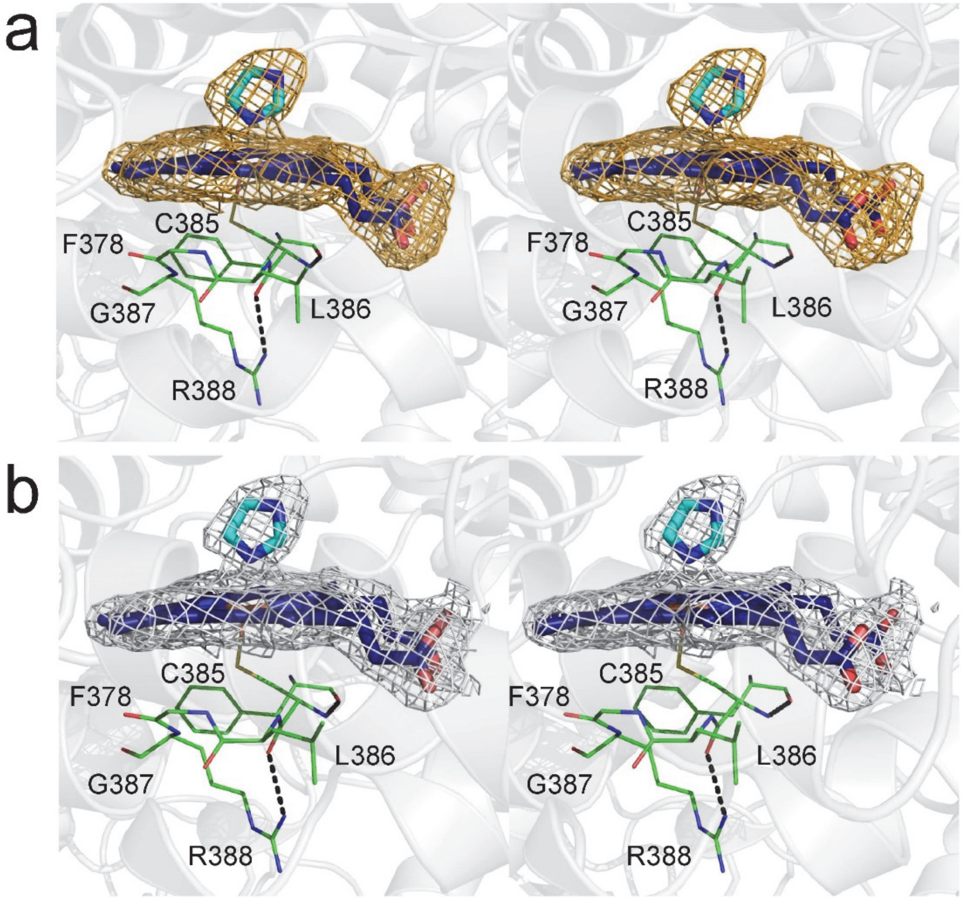

Supplementary Figure 4. Stereo views of heme-binding site of CYP116B46. (a) The F_o-F_c omit map and (b) 2F_o-F_c electron density map of heme and the imidazole in the CYP116B46 are contoured at 2.0 and 1.0 σ, respectively.

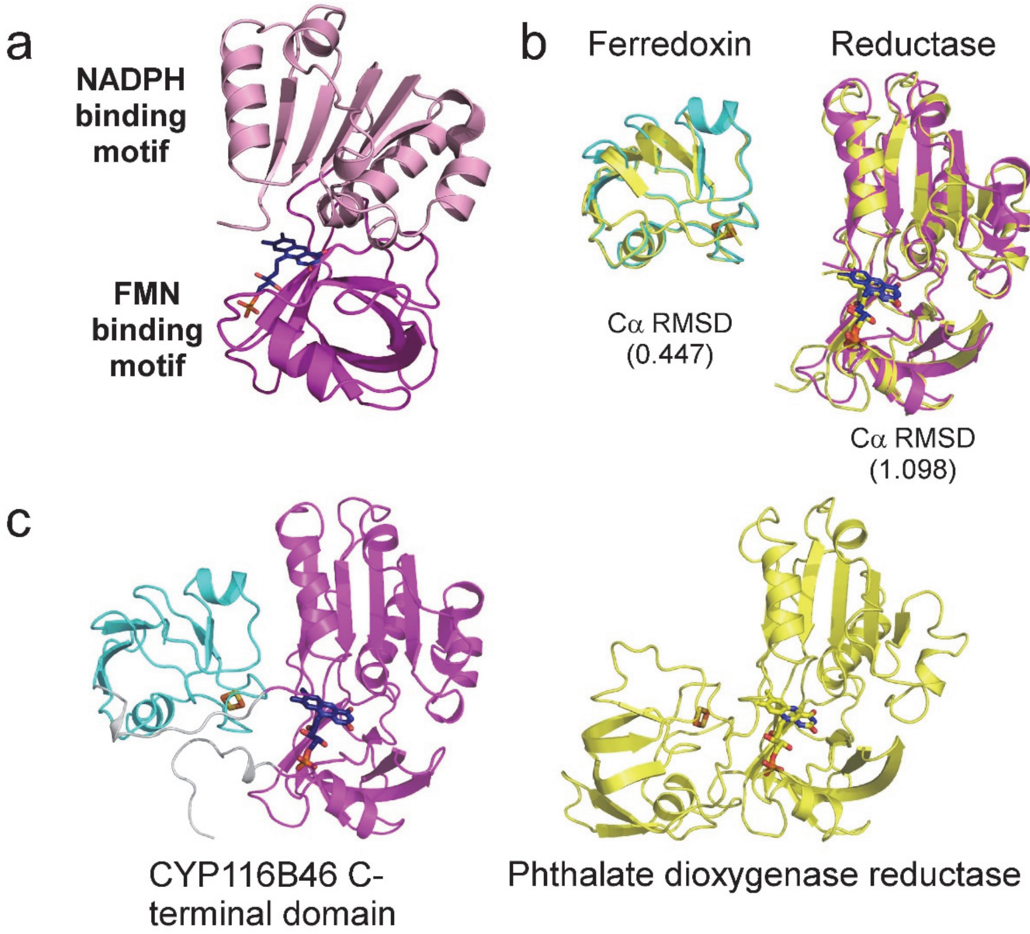

Supplementary Figure 5. C-terminal domain comparison. (a) The reductase domain of C-terminal region of CYP116B46 is presented in cartoon model, with NADPH- and FMN-binding motifs indicated. (b) Structural superimpositions of ferredoxin and reductase domains of phthalate dioxygenase reductase (yellow, PDB ID, 2PIA)[10] and CYP116B46 (cyan for ferredoxin, magenta for reductase domain). (c) Overall structures of C-terminal domain of CYP116B46 and phthalate dioxygenase reductase. Bound ligands are shown in sticks.

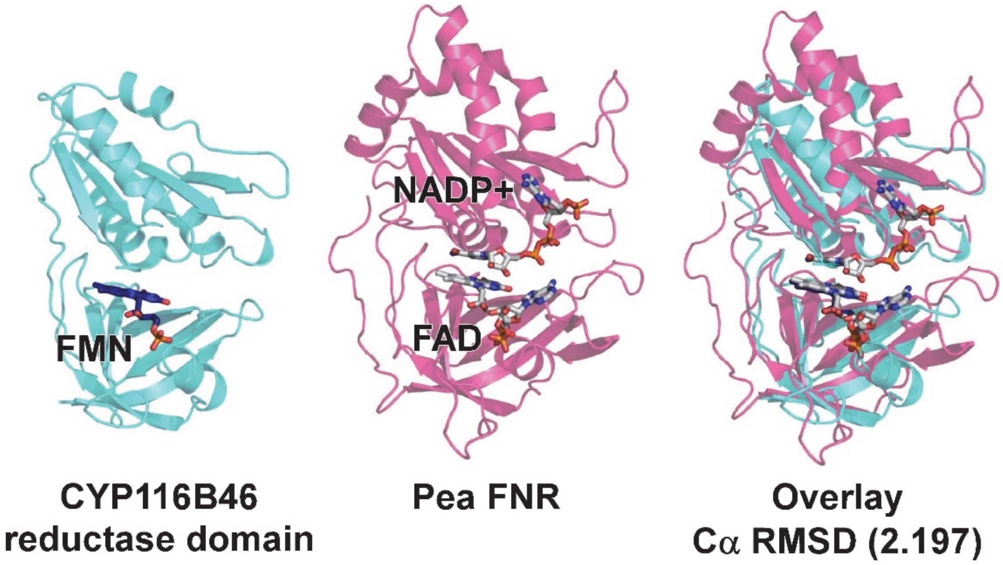

Supplementary Figure 6. Structure superimposition of CYP116B46 reductase domain and pea FNR. Crystal structures of CYP116B46 (cyan) reductase domain and FNR from pea (magenta, PDB ID, 1QFZ)[11] are presented in cartoon model, and the bound co-factors shown as sticks. The value of Cα RMSD is indicated in the parentheses below the superimposed structures with unit as Å.

Supplementary Table 1. Data collection and refinement statistics of CYP116B46 crystal.

	**CYP116B46**
Data collection	
Space group	*P4₃2₁2*
Unit-cell	
a, b, c [Å]	94.72, 94.72, 242.64
α /β /γ (°)	90/90/90
Resolution (Å)	25-2.13 (2.21-)
Unique reflections	62367 (6151)
Redundancy	16.5 (15.2)
Completeness (%)	99.7 (100.0)
Average I/σ (I)	32.5 (2.0)
CC 1/2	0.962 (0.823)
Refinement	
No. of reflections (work set)	62276 (5789)
No. of reflections (free set)	3114 (305)
R_{work} (95% data)	0.167 (0.243)
R_{free} (5% data)	0.210 (0.295)
Rmsd bonds (Å)	0.013
Rmsd angles (°)	1.216
Dihedral angles	
Most favored (%)	96.4
Allowed (%)	3.6
Disallowed (%)	0.0
No. Of non-H atom / average B [$Å^2$]	
Protein	6150/45.8
Water	147/42.2
Ligand	544/51.8
PDB code	**6LAA**

Values in parentheses are for the outermost resolution shells.

Supplementary Table 2. Sequence of synthetic gene of CYP116B46

ATGGAAACCGAACTGAAAGAAACCGCGCGTGGCACCTGCCCGGTTGCGCACGG
TGGCCAGTCTAGCGTTGGTGGTTGCCCGGTTCACCGTCTGGCGGAAGACTTTGA
TCCGTTCCAGGATGCGTACATGGCGGACCCGGCGCAATTTGTGCGCTGGGCGC
GTGAACAGGTTCCGATCTTTTATGCGCCTAAACTGAACTATTGGGTCGTTACCC
GCTACGACACCATCAAACAGATCTTCCGTGATCCGGTTACCTTCTCCCCGAGCA
ACGTGCTGCAGTCCTTTGCCCAGCCAAGCGCCGAAGTTCGCCAGGTTCTGGAA
CGTTATGGTTACGCTTTTAATCGTACCCTGGTTAACGAAGACGAACCGATGCAC
CTCGAACGTCGTCGTGTGCTGATGGAGCCGTTCGCTTCTGAACACCTCGCAGAA
CACGAACCAATGGTGCGTGAACTGGTTCGCCGCGCGGTGAACCGTTTTATTGA
CACTGGCCGTGCTGATCTGGTAGACCAGATGATTTGGGAAGTGCCGTTCACCGT
TGCGCTGCACTTCTTAGGTGTTGATGATGACGATCGTGAAAAAATGCGTCGTTT
CGCCATCGCACACACTGTTAACGCATTTGGTCGTCCGTCTCCGGAGGAACAGCT
GGCGGTGGCTGAAACGGTTGGCCAGTTCTGGCAGTTCTGTGGCGAAGTTCTGG
AAAAAATGCGCCGTACCGCAGACGGTCCAGGTTGGATGCGCTATAGTATCCGT
CAGCAGAAACTGTACCCGGACGTTGTGACCGACAGCTACCTGCACAGCATGAT
GCAGGCGATCATCGTGGCAGCCCACGAAACCACCGTTTTCGCGACCACCAACG
CCTTGAAAACCCTGCTGGAACACGAAACCGTGTGGCGTGAAATTTGCGCGGAT
CCGTCGCTGATCCCGGCAGCTGCAGAAGAATGCCTGCGCTACAATGGCCCGGT
GGCAGGTTGGCGCCGTCGTACCACCCGTGAAGTTGAGGTTGAAGGCGTCCGTC
TGCCGGTTGGCGCGAACATCCTGATGGTGGTTGCATCTGCGAACCATGATTCTG
CGCATTTCGATGATCCTGAGTTCTTCGATATTGGCCGTAGCAACGCGAGCGAAC
ACCTGAACTTCGGCTATGGCGCCCACCAGTGCCTGGGCCGCAACTTGGGTCGT
ATGGAAATGCAGATCATGATCGAAGAACTGTCCCGTCGCTTACCGCACATGCG
CCTGGCCGAACAGCGTTTCGACTATCTGCACAACGTTTCCTTCCGTGCGCCACG
CCACCTGTGGGTCGAATGGGACCCGGCGCAGAATCCGGAACGTCGCGATCCGG
ACATTCTGCGCCTGCGTCAGCCAGTACGCATCGGTCCACCGCGTGCGAAAGAC
GTTGTGCGCACCATGGAAGTTGCTGCAGTTGAACGCCCGTCCGAAGATATCGT
GGTTCTGCACCTGACCCGCCCGGACCGTCGTCCGCTGCCGCGTTGGTCTCCGGG
CGCTCATATCGATATTGAATGTGGCGAACCTGATCGTTCCCGCCAGTATAGCCT
GTGCTCTGACCCAGAAAACCGTGACGCATGGCGTGTAGCGGTACAGCGTGACC
CGGCGAGCCGCGGCGGCTCTCGCTGGATTCACGAAGAGGTGCGTCCGGGTATG
CTGCTGCGCGTTCGTGGTCCGCGTAATTCCTTCCGTCTGGACGAACACGCTCCG
CGTTACCTGTTCCTGGCGGGTGGCATCGGCATCACCCCGATCATGACTATGGCG
GCGCGCGCGAAAGAGCTGGGTACCGATTACGAACTGCATTATTCTGTGCGTTCT
CGCACCAGCCTGATCTTCGTGGATGAACTGCGCCAGATCCACGGTGATCGCCT
GCACGTGTACGTGAGCGAAGAAGGTGTGCGTAACGACCTGGCAGCGCTGATTC
GCCGTGCGAGCGCTGGCACCCAGATCTACGCATGTGGCCCGCAGCGTATGCTG
GACACCCTGGAACGCCTGATCGAAAACCGTCCGGAAGTAACCCTGCGTGTGGA
ACATTTCTTCGGCGAACCGAGCCACCTGGATCCGGCGAAAGAACGTCCGTTCC
AGGTTGTGCTGCGTAACTCTGGTCTGACCGTTGAAGTTCCGGCCGATAAAACCC
TGTTGGAAGTTTTGCGTGCATACAACATCGAAGTGCAGTCTGATTGTGAAGAA
GGTCTGTGCGGCACTTGTGAAGTTTCTGTTGTTGAAGGTGAAGTTGATCACCGT
GATTCCGTGCTGACCCGCGCAGAACGTCGTGAAAACCGTCGTATGATGTGCTG
TTGCTCTCGTGCTAAAACCGAACGTCTGGTTCTGGACCTGTAA

Supplementary Table 3. Mutagenesis oligonucleotides

	Sequence (5'→3') (mutation sites are underlined)
F378A-F	GAAC**GCT**GGCTATGGCGCCCACCA
F378A-R	CCATAGCC**AGC**GTTCAGGTGTTCGC
R388A-F	GTGCCTGGGC**GCC**AACTTGGGTCGTATGGAAATGCAGAT
R388A-R	GACCCAAGTT**GGC**GCCCAGGCACTGGTGGGCGCCATA
R392A-F	CAACTTGGGT**GCC**ATGGAAATGCAGATCATGATCGAAGAACTGTCCCGT
R392A-R	GCATTTCCAT**GGC**ACCCAAGTTGCGGCCCAGGCACTGGT
R718A-F	GGAAGTTTTG**GCT**GCATACAACATCGAAGTGCAGTCTGATT
R718A-R	TGTTGTATGC**AGC**CAAAACTTCCAACAGGGTTTTATCGGCC
E723A-F	ATACAACATC**GCA**GTGCAGTCTGATTGTGAAGAAGGTCTGTGCGGCACTT
E723A-R	TCAGACTGCAC**TGC**GATGTTGTATGCACGCAAAACTTCCAACAGGGTTTTAT
Q725A-F	CATCGAAGTG**GCG**TCTGATTGTGAAGAAGGTCTGTGCGGCACTT
Q725A-R	CACAATCAGA**CGC**CACTTCGATGTTGTATGCACGCAAAACTTCCAACAGG
S726A-F	TGCAG**GCG**GATTGTGAAGAAGGTCTGT
S726A-R	ACAGACCTTCTTCACAATC**CGC**CTGCA
E729A-F	TCTGATTGT**GCA**GAAGGTCTGTGCGGCACTTGTGAAGTT
E729A-R	ACAGACCTTC**TGC**ACAATCAGACTGCACTTCGATGTTGT

Supplementary References

1. Sevrioukova IF, Li H, Zhang H, Peterson JA, Poulos TL. Structure of a cytochrome P450–redox partner electron-transfer complex. *Proceedings of the National Academy of Sciences* **96**, 1863-1868 (1999).

2. Wang M, Roberts DL, Paschke R, Shea TM, Masters BS, Kim JJ. Three-dimensional structure of NADPH-cytochrome P450 reductase: prototype for FMN- and FAD-containing enzymes. *Proc Natl Acad Sci U S A* **94**, 8411-8416 (1997).

3. Hamdane D, Xia C, Im SC, Zhang H, Kim JJ, Waskell L. Structure and function of an NADPH-cytochrome P450 oxidoreductase in an open conformation capable of reducing cytochrome P450. *The Journal of biological chemistry* **284**, 11374-11384 (2009).

4. Aigrain L, Pompon D, Morera S, Truan G. Structure of the open conformation of a functional chimeric NADPH cytochrome P450 reductase. *EMBO reports* **10**, 742-747 (2009).

5. Hiruma Y*, et al.* The structure of the cytochrome P450cam-putidaredoxin complex determined by paramagnetic NMR spectroscopy and crystallography. *Journal of molecular biology* **425**, 4353-4365 (2013).

6. Tripathi S, Li H, Poulos TL. Structural basis for effector control and redox partner recognition in cytochrome P450. *Science (New York, NY)* **340**, 1227-1230 (2013).

7. Strushkevich N, MacKenzie F, Cherkesova T, Grabovec I, Usanov S, Park HW. Structural basis for pregnenolone biosynthesis by the mitochondrial monooxygenase system. *Proc Natl Acad Sci U S A* **108**, 10139-10143 (2011).

8. Tavanti M, Porter JL, Levy CW, Gomez Castellanos JR, Flitsch SL, Turner NJ. The crystal structure of P450-TT heme-domain provides the first structural insights into the versatile class VII P450s. *Biochem Biophys Res Commun* **501**, 846-850 (2018).

9. Ciaramella A, Catucci G, Gilardi G, Di Nardo G. Crystal structure of bacterial

CYP116B5 heme domain: New insights on class VII P450s structural flexibility and peroxygenase activity. *International Journal of Biological Macromolecules* **140**, 577-587 (2019).

10. Correll CC, Batie CJ, Ballou DP, Ludwig ML. Phthalate dioxygenase reductase: a modular structure for electron transfer from pyridine nucleotides to [2Fe-2S]. *Science (New York, NY)* **258**, 1604-1610 (1992).

11. Deng Z*, et al.* A productive $NADP^+$ binding mode of ferredoxin-$NADP^+$ reductase revealed by protein engineering and crystallographic studies. *Nature structural biology* **6**, 847-853 (1999).
